# Host Specificity And Co-Speciation In Avian Haemosporidia In The Western Cape, South Africa

**DOI:** 10.1371/journal.pone.0086382

**Published:** 2014-02-03

**Authors:** Sharon Okanga, Graeme S. Cumming, Philip A. R. Hockey, Lisa Nupen, Jeffrey L. Peters

**Affiliations:** 1 Percy FitzPatrick Institute, A Centre of Excellence for the Department of Science and Technology and National Research Foundation, University of Cape Town, Cape Town, South Africa; 2 Department of Biological Sciences, Wright State University, Dayton, Ohio, United States of America; Indian Institute of Science, India

## Abstract

Host and pathogen ecology are often closely linked, with evolutionary processes often leading to the development of host specificity traits in some pathogens. Host specificity may range from ‘generalist’, where pathogens infect any available competent host; to ‘specialist’, where pathogens repeatedly infect specific host species or families. Avian malaria ecology in the region remains largely unexplored, despite the presence of vulnerable endemic avian species. We analysed the expression of host specificity in avian haemosporidia, by applying a previously developed host specificity index to lineages isolated from wetland passerines in the Western Cape, South Africa. Parasite lineages were isolated using PCR and identified when possible using matching lineages deposited in GenBank and in MalAvi. Parasitic clades were constructed from phylogenetic trees consisting of *Plasmodium* and *Haemoproteus* lineages. Isolated lineages matched some strains of *Plasmodium relictum*, *P. elongatum*, *Haemoproteus sylvae* and *H. lanii*. *Plasmodium* lineages infected a wide range of hosts from several avian families in a generalist pattern of infection. *Plasmodium* spp. also exhibited an infection trend according to host abundance rather than host species. By contrast, *Haemoproteus* lineages were typically restricted to one or two host species or families, and displayed higher host fidelity than *Plasmodium* spp. The findings confirm that a range of host specificity traits are exhibited by avian haemosporidia in the region. The traits show the potential to not only impact infection prevalence within specific host species, but also to affect patterns of infection at the community level.

## Introduction

Recent advances in molecular ecology have profoundly altered the nature of research on parasites and parasitism. For many parasitic microorganisms, such as malaria parasites, molecular ecology has provided both a newfound ability to identify parasites accurately and the tools for understanding how and when host-parasite coevolution occurs. Within the broader context of host-parasite relationships, one of the more intriguing questions that molecular methods have opened up is that of how differences in the degree of host specialization in closely related parasites have arisen. For example, avian haemosporidia (some of which cause avian malaria), occupy niches that range from extreme host specialization to extreme host generalization. Each clade represents a persistent [hence, successful] solution to a complex problem that incorporates environmental influences as well as elements of parasite, vector, and host ecology and physiology. Unravelling the roles of these different elements in host-parasite coevolution has the potential to offer profound insights not only into parasite ecology, but also more generally into the process of speciation in heterogeneous habitats and communities.

The likelihood that a potential host will come into contact with a parasite is influenced by its ecological traits, including such variables as its life history, movement patterns, territoriality, diet, social behaviours, and overall abundance as well as the broader community to which it belongs [1],[2]. These traits, and the internal environment offered by a potential host, may be differentially suited to the life history and needs of a particular parasite. In highly selective parasites (specialists), success is closely linked to the availability and distribution of a single preferred host species, often resulting in disease prevalence being an indirect reflection of that host's ecology [3],[4],[5]. In the converse scenario, generalism, a parasite's success is dependent upon its ability to infect as wide a variety of hosts as possible. Hence, the wider the choice of hosts available, the greater the chances of success for a highly generalist parasite, especially in biodiverse environments [6]. The number of hosts a parasite infects is often a manifestation of specific life history traits resulting from the co-evolution of host and parasite [7],[8]. Generally, parasites infecting a smaller group of hosts or closely related host species have often developed a more long-term co-evolutionary history with their hosts. The number of hosts a parasite infects – also referred to as ‘host specialization’- can potentially be defined using the phylogenetic distances between host species per parasite [8].

Prior research on the host specificity of avian haemosporidia has revealed a considerable amount of elasticity amongst avian haemosporidia regarding their level of host specificity. *Plasmodium* and *Haemoproteus* spp. can display alternating specialist and generalist traits, with some parasites infecting a wide range of avian hosts and showing greater potential to invade novel hosts [9]. Some studies have concluded that particular avian haemosporidia show little evidence for host specificity [10],[11], whereas other studies show parasites exhibiting greater selectivity [4],[12],[13]. Similarly, some haemosporidia can infect multiple species, but predominantly infect only a select few hosts – a trait that cannot be said to be purely specialist but shows preferential tendencies [14],[15]. Partial specialization, which has also been demonstrated for African ticks [16], is often seen when there is more than one preferred host and/or when hosts are abundantly available [17].

Results from previous studies [13],[18] provide some indication of the richness and host specificity of avian haemosporidian lineages occurring in Africa but shed little light on evolutionary mechanisms. As a step towards enhancing knowledge on host-parasite interactions in avian haemosporidia and potential evolutionary relationships [18],[19], we analysed patterns of infection and phylogenetic links between *Haemoproteus* and *Plasmodium* parasites and their avian hosts, across 26 wetland-associated passerine bird communities in the Western Cape of South Africa. As the topic in this region is poorly studied, we further aimed to establish whether trends of host specificity were general to both *Plasmodium* and *Haemoproteus* or restricted to one genus or another. We also aimed to determine whether observed trends of host specificity were common to an avian family, or specific to individual species.

## Materials And Methods

### Ethics Statement

This research was carried out in strict accordance with the recommendations given by the UCT Science Faculty Animal Ethics Committee (University of Cape Town). The research was conducted after approval from the UCT Science Faculty Animal Ethics Committee. This research did not involve the sampling of endangered or protected species. Approval for this research and access to field sites was granted by private landowners in the Western Cape and the City of Cape Town. Research permits granting access to protected areas were issued by SANParks (South African National Parks Board) and by Cape Nature (the Western Cape Nature Conservation Board).

### Sampling Sites

**Figure 1:**
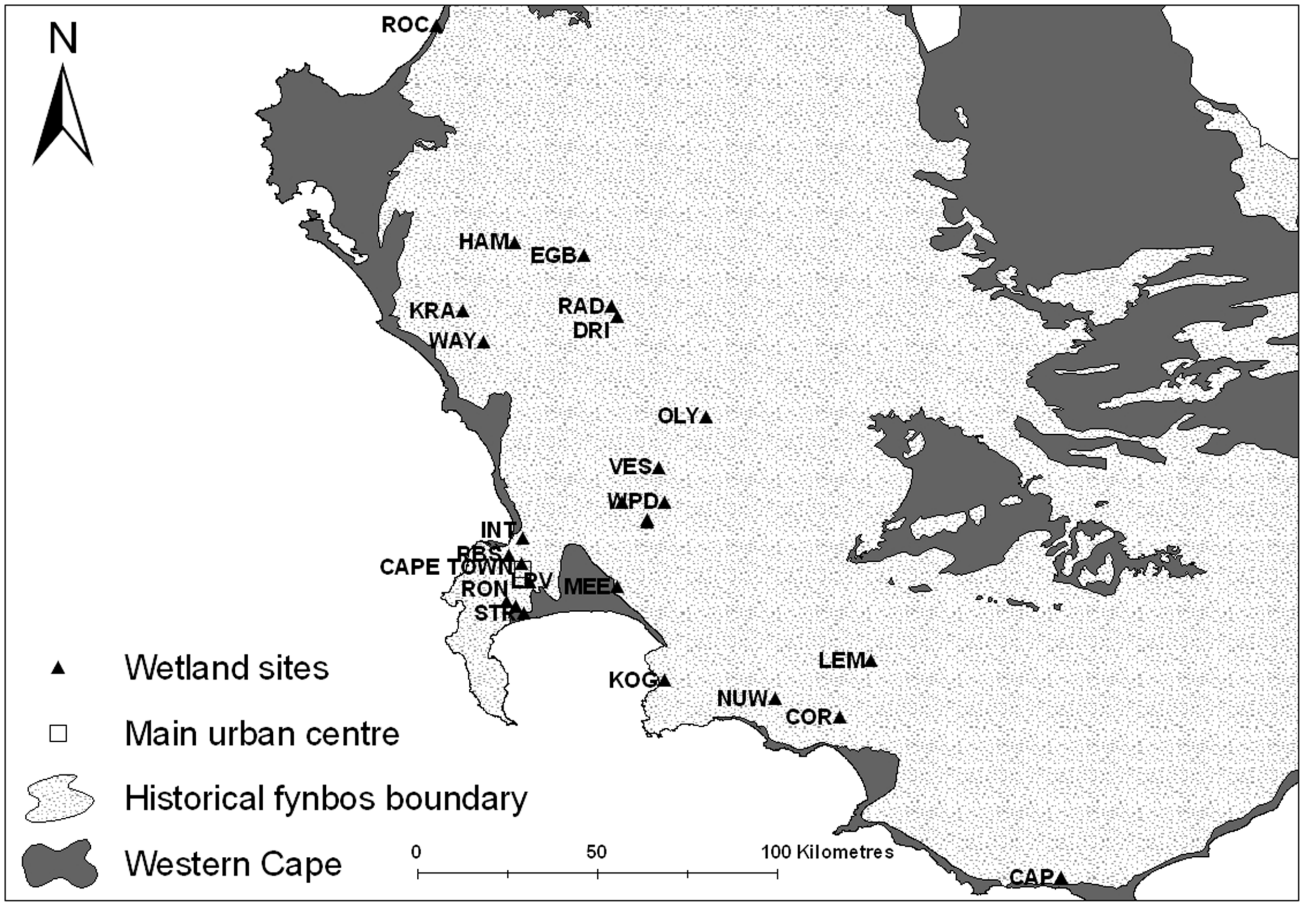
Wetland sampling sites in the Western Cape.

Sampling took place at 26 wetlands located in the Western Cape (Fig. 1). The chosen wetlands provide key habitats for a large variety of birds, and also provide excellent breeding conditions for the vectors of avian haemosporidia [20],. The region is currently devoid of human malaria, but has a history of avian malaria infection [18],[22],[23]. Sites were located within the historical boundaries of the fynbos biome of the Western Cape, where development has fragmented the biome into habitats varying from densely urban, to agricultural, to near pristine. Sites were visited twice over the course of two years (2010 and 2011), with samples collected at each site once in summer (January to March) and once in winter (July to September).

### Sampling

Birds were trapped using mist nets. Wetland passerines were the target sample group, although birds from other families were also sampled when caught. Sampling was conducted by pricking the brachial vein and collecting blood into a capillary tube [24], which was then preserved in vials containing 10% SDS lysis buffer. The vial was sealed and the sample stored for molecular processing. All sampled birds were ringed (to identify individuals and future recaptures) and released after sampling.

### Molecular Analysis

Blood samples were analysed using PCR, as detailed by Cumming et al. [13]. DNA was extracted from whole blood using the DNeasy tissue kit (Qiagen). A nested PCR was conducted to amplify and sequence 478 bp of the mitochondrial cytochrome *b* gene following an adapted protocol of Waldenström et al. [25]. The protocol used 8 µL of water, 12.5 µL of GoTaq Green Master Mix (Promega, Madison, WI, USA), 1.5 µL of primer HaemNF (10 nM concentration – [25]), 1.5 µL of primer HaemNR2 (10 nM concentration), and 2 µL of sample in a 25 µL reaction. The PCR profile was conducted as follows: 94°C for 3 minutes, 35 cycles of 94°C for 30 seconds, 50°C annealing for 30 seconds, and 72°C extension for 45 seconds, and finally a 72°C step for 10 minutes. A second nested PCR was then carried out with 1.5 µL of the first PCR product using the nested primers HaemF (5′- AATGGTGCTTTCGATATATGCATG-3′) and HaemR2 (5′-GCATTATCTGGATGTGATAATGGT-3′), as per Bensch et al. [9], using the same reaction mix as above. The nested PCR profile was identical to that of the first PCR. Gel electrophoresis was used to test for the presence of a PCR product indicative of a positive infection. PCR products were cleaned with 1.8 µL of Agencourt Ampure XP beads (Beckman Coulter, Inc., Indianapolis, IN, USA) per 1 µL of PCR product, 200 µL of 70% ethanol, and 40 µL of water. Two PCR trials were conducted for each sample under identical conditions. PCR products were sequenced using the BigDye v3.1 Cycle Sequencing Kit (Life Technologies, Grand Island, NY, USA) in a reaction with 1 µL of BigDye, 1.75 µL of 5× buffer, 1 µL of primer (10 nM concentration), and 4.5 µL of water. The final products were sent to the DNA Sequencing Facility on Science Hill at Yale University, New Haven, CT, USA, for automated sequencing on an ABI 3730× (Applied Biosystems, Foster City, CA, USA). This PCR routine only detected parasites within the *Plasmodium* and *Haemoproteus* genera, as these primers do not amplify *Leucocytozoon* [25]. Forward and reverse sequences were aligned and edited using Sequencher v5.0 (Gene Codes Corporation, Ann Arbor, MI, USA).

To identify parasitic strains, sequences were individually entered into GenBank® and MalAvi (a database specific to avian haemosporidia lineages). Searches for matching sequences were run using BLAST (Basic Local Alignment Search Tool) to identify the genus and species (when possible) of the lineages infecting the host. All matching and closely related sequences were added to the dataset, then edited and aligned using BioEdit Sequence Alignment Editor [26]. MEGA 5.0 (Molecular Evolutionary Genetics Analysis – [27]) was used to conduct genetic analyses, to choose the most appropriate model of evolution, and to construct maximum likelihood (ML) phylogenetic trees. In addition, a Bayesian analysis was run in MR Bayes for 1 million generations with a burn-in of 250 000 generations, sampling one in every 100 trees [28],[29]. The general time reversible model with a discrete gamma distribution (GTR+G; G = 0.20) received the best Bayesian Information Criterion score and was applied to both the ML and Bayesian phylogenetic analyses. The tree was rooted on the branch connecting *Plasmodium* and *Haemoproteus* clades. Node support was evaluated using 1000 bootstrap replicates, with bootstrap values greater than 50% used for the final tree [30]. Sample sequences emerging in the same branches as GenBank and/or MalAvi database sequences were assumed to be the same species.

### Statistical Analysis

Only successfully sequenced samples were used in statistical analysis, as the genera for positives with failed sequencing could not be determined. Samples emerging from the same branches in the phylogenetic trees were taken as the same species and collectively referred to as ‘clades’. Each clade in the phylogenetic trees was given a code corresponding to A – I (*Plasmodium* lineages) and J – S (*Haemoproteus* lineages). Birds were grouped according to their families as classified in the *Roberts - Birds of Southern Africa* ([31] – see Table 1).

**Table 1:** Sampled avian families detailing species sampled from each family.

Family	Sampled species
**Cisticolidae**	Levaillant's Cisticola (*Cisticola tinniens*); Karoo Prinia (*Prinia maculosa*); Neddicky (*Cisticola fulvicapillus*)
**Estrildidae**	Common Waxbill (*Estrilda astrild*); Swee Waxbill (*Estrilda melanotis*)
**Hirundinidae**	Brown-throated Martin (*Riparia paludicola*); White-breasted Swallow (*Hirundo albigularis*); Greater Striped Swallow (*Hirundo cucullata*)
**Laniidae**	Common Fiscal (*Lanius collaris*)
**Motacillidae**	Cape Wagtail (*Motacilla capensis*)
**Muscicapidae**	Cape Robin-Chat (*Cossypha caffra*); Fiscal Flycatcher (*Sigelus silens*); Karoo Scrub Robin (*Cercotrichas coryphaeus*)
**Nectariniidae**	Orange-breasted Sunbird (*Nectarinia violacea*); Southern Double-collared Sunbird (*Nectarinia chalybea*)
**Passeridae**	Cape Sparrow (*Passer melanurus*); House Sparrow (*Passer domesticus*)
**Ploceidae**	Cape Weaver (*Ploceus capensis*); Southern Masked Weaver (*Ploceus velatus*); Southern Red Bishop (*Euplectes orix*); Yellow Bishop (*Euplectes capensis*)
**Promeropidae**	Cape Sugarbird (*Promerops cafer*)
**Sylviidae**	African Reed Warbler (*Acrocephalus baeticus*); Lesser Swamp Warbler (*Acrocephalus gracilirostis*); Little Rush Warbler (*Bradypterus baboecala*)
**Zosteropidae**	Cape White-eye (*Zosterops virens*)

Expected infection prevalences for bird families were calculated as : (Σ (Inf_i_/S_f_))×Inf_f_,

Where Inf_i_ = sum of infected birds; S_f_ = total individuals sampled in infected families; Inf_f_ = infected individuals per family. This formula was also used to compute expected infection prevalence for parasitic clades as: (Σ(Inf_hp_/S_i_))×Inf_c_.

Where Inf_hp_ = sum of infected birds per genus (*Plasmodium* or *Haemoproteus*); S_i_ = total individuals infected; Inf_c_ = infected individuals per clade.

We compared expected and observed prevalence between infected families and between generic clades using chi-squared tests (*x* ^2^). We expected that potential ‘preferred’ host species would belong to families with heavier than expected infection prevalences. A one-way ANOVA was employed to test for any significant differences between the prevalence of infected species and the mean number of birds infected per parasitic clade. We explored infection trends between parasitic clades and infected avian families using Pearson's product moment correlations (*r*) and ordination by canonical correspondence analysis (CCA) conducted following procedures within the vegan 2.0-2 package created for R [32]. For CCA, two matrices were created grouping birds across sites—one was structured according to numbers infected per family, and the other according to numbers infected per clade (mixed infections were treated as two separate infections to consolidate infections to one genus or the other). Each matrix was standardized to: a) make them comparable (by subtracting the mean and dividing by the standard deviation) before being run through CCA ordination procedures, and b) to avoid the outcome being biased from one single variable.

We quantified host specificity for each parasite lineage in our study by applying two indices describing the taxonomic distinctness (S_TD_) and diversity (VAR_STD_) of infected hosts per lineage [8]. S_TD_ was calculated as: S_TD_ = 2ΣΣ w*_ij_*/s(s−1); where *w* = index score between species *i* and *j* (e.g., w = 1 for species pairs in the same genus, and w = 4 for species in the same class but different order); and *s* = the total number of infected species.

VAR_STD_ is complementary to S_TD_, providing an indication of phylogenetic diversity between host species. VAR_STD_ was calculated as: VAR_STD_ = ΣΣ (w*_ij_*−S_TD_)^2^/s(s−1).

S_TD_ and VAR_STD_ were calculated with the inclusion of other host species sourced from GenBank, to give a broader idea of parasite lineage specificity. These indices were not calculated for clades infecting a single species [8].

## Results

### Infection Prevalence By Host Family

Out of 974 birds tested, a total 218 samples (22%) were identified as positive for malarial infections (Table 2). Twenty species from 12 families were infected. Observed and expected prevalence rates differed significantly amongst infected families (*x^2^* = 22.45; d. f. = 11; *p* = 0.021). The Ploceidae exhibited a significantly higher than expected infection prevalence (*p*<0.001), whereas prevalences within other families generally were lower than expected (Table 3).

**Table 2:** *Plasmodium* and *Haemoproteus* spp. prevalence amongst host species; the number of lineages per species is shown in brackets.

Infected Species	Sampled	Infected	% *Plasmodium*	% *Haemoproteus*
African Reed Warbler (*Acrocephalus baeticatus*)	16	3	6 (1)	13 (2)
Brown-throated Martin (*Riparia paludicola*)	7	1	14 (1)	0
Cape Robin-Chat (*Cossypha caffra*)	40	7	13 (3)	5 (2)
Cape Sparrow (*Passer melanurus*)	19	4	11 (1)	11 (1)
Cape Sugarbird (*Promerops cafer*)	12	1	8 (1)	0
Cape Wagtail (*Motacilla capensis*)	8	2	25 (1)	0
**Cape Weaver (** ***Ploceus capensis*** **)**	**208**	**60**	**27 (5)**	**1 (1)**
Cape White-eye (*Zosterops virens*)	30	5	4 (1)	15 (1)
Common Fiscal (*Lanius collaris*)	9	4	22 (1)	22 (1)
Common Waxbill (*Estrilda astrild*)	31	1	3 (1)	0
Fiscal Flycatcher (*Sigelus sigens*)	4	1	25 (1)	0
House Sparrow (*Passer domesticus*)	2	1	0	50 (1)
Karoo Prinia (*Prinia maculosa*)	11	2	18 (2)	0
Karoo Scrub Robin (*Cercotrichas coryphaeus*)	1	1	100 (1)	0
Lesser Swamp Warbler (*Acrocephalus gracilirostris*)	52	1	2 (1)	0
Levaillant's Cisticola (*Cisticola tinniens*)	44	2	2 (1)	2 (1)
Little Rush Warbler (*Bradypterus baboecala*)	11	1	9 (1)	0
Southern Double-collared Sunbird (*Cinnyris chalybeus*)	6	1	17 (1)	0
Southern Masked Weaver (*Ploceus velatus*)	135	30	22 (3)	0
**Southern Red Bishop (** ***Euplectes orix*** **)**	**195**	**76**	**30 (7)**	**9 (4)**
Yellow Bishop (*Euplectes capensis*)	83	14	16 (3)	1 (1)
**Total**	**924**	**218**	**182 (18%)**	**36 (4%)**

Species in bold had higher than expected infection prevalences for either *Plasmodium* and/or *Haemoproteus*.

**Table 3:** Variance in observed and expected values amongst infected host families; (-) indicates no difference in values and significant deviance is indicated by *p*<0.05^*^ and *p*<0.01^**^.

Family	Observed infections	Expected infections	*x^2^*
Cisticolidae	4	13	36.81^**^
Estrildidae	1	7	25.44^**^
Hirundinidae	1	3	4.75^*^
Laniidae	4	2	2.17
Motacillidae	2	2	-
Muscicapidae	9	11	4.03^*^
Nectariniidae	1	2	0.50
Passeridae	5	5	-
Ploceidae	180	144	122.09^**^
Promeropidae	1	3	4.36^*^
Sylviidae	5	18	57.11^**^
Zosteropidae	5	6	0.70
**Total**	**218**	**216**	

Bold text denotes a significantly heavier than expected infection.

**Figure 2:**
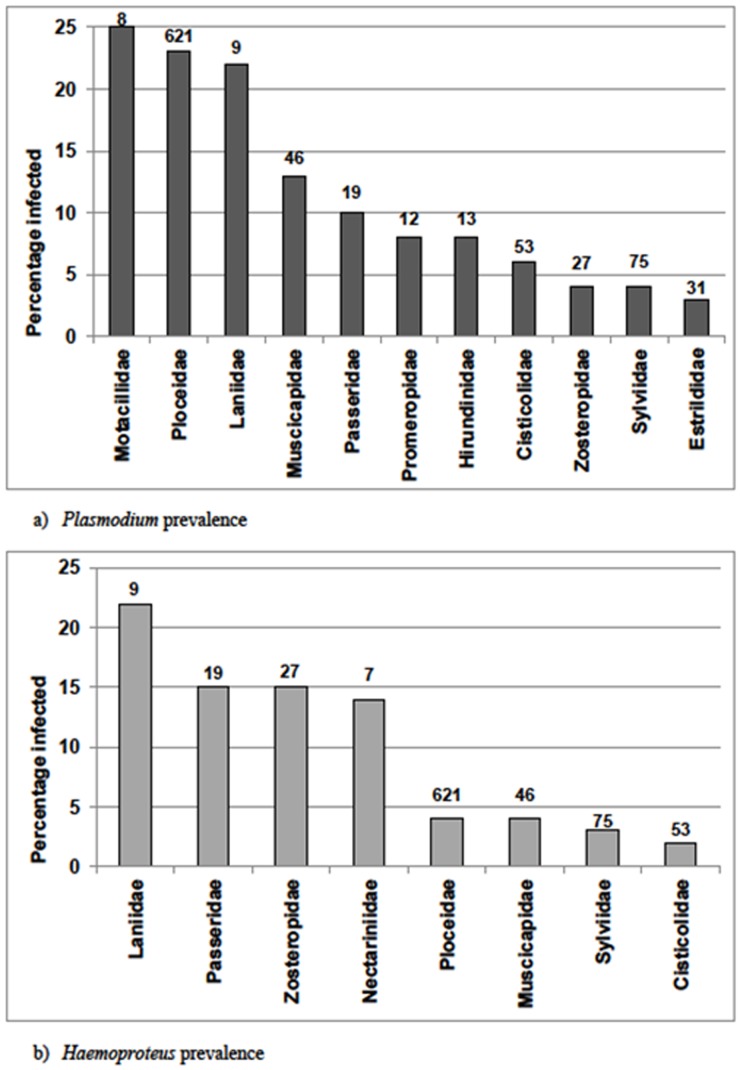
Variations in *Plasmodium* and *Haemoproteus* spp. prevalence amongst infected avian families. Number of birds sampled per family is indicated above each column (*Plasmodium*: n = 913; *Haemoproteus*: n = 857).

*Plasmodium* spp. accounted for the majority of infections. One hundred and eighty two birds (19%) from twelve families were infected with prevalences varying between 3% and 26% among species. *Plasmodium* was the only genus infecting Motacillidae, Promeropidae, Hirundinidae and Estrildidae (Fig. 2). *Plasmodium* infections were also notably higher in the Laniidae, Ploceidae and Motacillidae, with prevalence ranging from 23% to 25%. *Haemoproteus* spp. infected 36 birds (4%) from eight families and varied from 2% to 22% infection prevalence among species. Infection prevalence was highest in the Laniidae, Passeridae and Zosteropidae, with heaviest prevalences ranging between 15% and 22%.

### Host Species Infection Prevalence

Twelve host species exhibited infections from only one parasitic genus (Table 2), whereas Common Fiscals, Cape Sparrows and Levaillant's Cisticola had equal prevalences of both *Plasmodium* and *Haemoproteus*. Infection prevalences between individual species also differed significantly. Although Southern Red Bishops were the most heavily infected species, infection prevalences were lower than expected for both *Plasmodium* (*x^2^* = 8.27; *p*<0.005) and *Haemoproteus* (*x^2^* = 69.57; *p*<0.001). Southern Red Bishops were also infected with the richest diversity of parasite lineages (no. of lineages = 11), much higher than the mean of 2.43 (*x^2^* = 18.48; *p*<0.001). Similarly, Cape Weavers also displayed a lower than expected *Plasmodium* prevalence (*x^2^* = 9.43; *p*<0.005) and *Haemoproteus* prevalence (*x^2^* = 36.16; p<0.001) as well as a larger diversity of parasite lineages (no. of lineages = 6; *x^2^* = 8.78; *p*<0.005).

**Figure 3:**
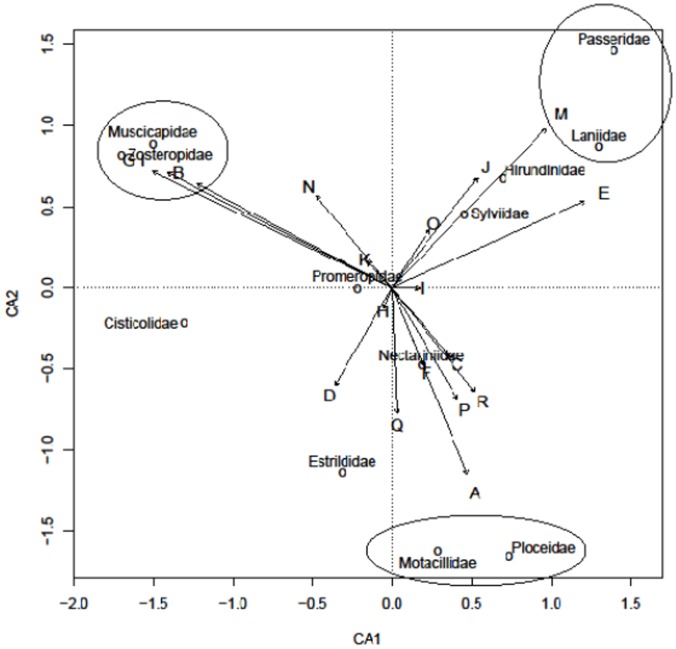
CCA scaling ordination bi-plot. Displayed are weighted correlations between *Plasmodium*/*Haemoproteus* clades (indicated by clade letters in front of divergent arrows) and avian family groups. Eigenvalues on axes and arrow lengths act as an approximate guideline to the strength of correlations and highlights the infection trends between parasitic clades and heavily infected family groups (circled).

Some parasite lineages displayed prominent infection trends in various host families (Fig. 3). *Plasmodium* lineages prominently infected the Ploceidae (Clades A and C) and Motacillidae (D) in particular (encircled in Fig. 3). The Ploceidae were also predominantly infected with *Haemoproteus* lineages (Clades P, Q and R); otherwise *Haemoproteus* infections occurred mainly in the Zosteropidae (L), Laniidae (M) and Passeridae (N and O).

### Haemosporidian Parasite Trends Of Infection

**Figure 4:**
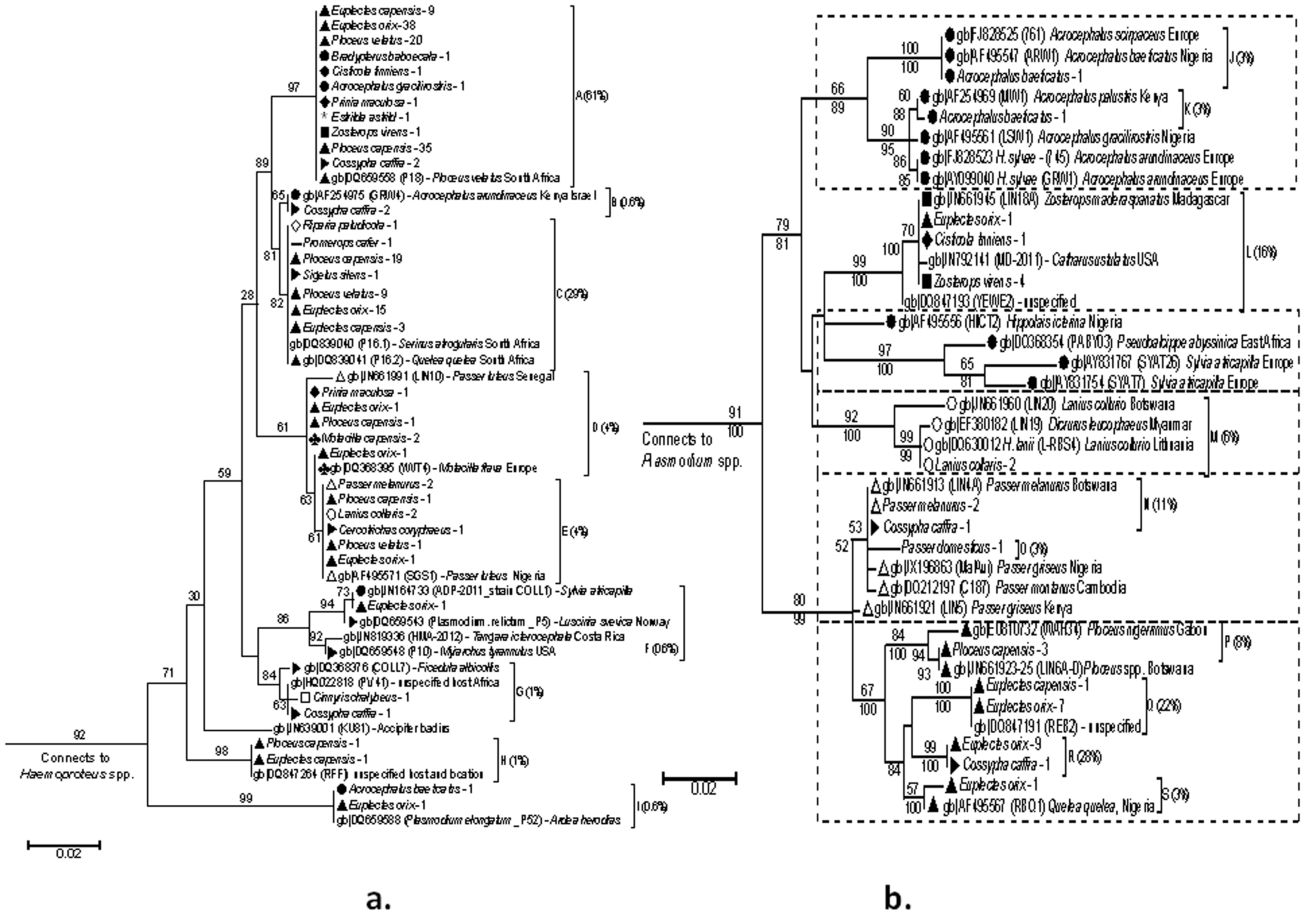
**Fig. 4a** *Plasmodium* phylogenetic tree with bootstrap values (>50%) displayed. Nodal support values from maximum likelihood analysis are displayed above branches and those from Bayesian analysis are below. Letters identify the clade (n = 9) with clade infection prevalence in brackets (n = 218). Samples are labelled by host *latin name* - number infected. GenBank strains are labelled by accession number (lineage code) – host latin name and location sampled. Avian families are represented by the following symbols: ▴Ploceidae; •Sylviidae; **▸**Muscicapidae; ▪Zosteropidae; ΔPasseridae; ♣Motacillidae; ○Laniidae; *Estrildidae; ♦Cisticolidae; ◊Hirundinidae; ⁃Promeropidae; □Nectariniidae. Other samples are species from uninfected or unrepresented families, or are samples of undefined origin. **Fig. 4b**. *Haemoproteus* phylogenetic tree with bootstrap values (>50%) displayed. Figures from maximum likelihood analysis are displayed above branches, whilst figures from Bayesian analysis are below. Coding for avian families is as for Fig. IVa (clades = 7). Blocks emphasize apparent patterns of lineage separation by host family group (n = 7).

Sample sequences branched into 19 clades—nine *Plasmodium* (Fig. 4a) and ten *Haemoproteus* (Fig. 4b). An average of 11.1 (±22.3) individual birds was infected per clade, with no significant variation from the mean (*F*_1, 16_ = 1.77; *p* = 0.20). *Plasmodium* lineages infected 18 species from all 12 infected families. A and C were the two most commonly occurring *Plasmodium* clades, infecting significantly more birds than all other clades combined (*x^2^* = 175.92; *p*<0.001). A significant variance occurred in bird species infected per clade (*F*_1, 16_ = 66.94; *p*<0.001) with Clades A and C infecting the widest range of host species and families. Clades A and C matched lineages described as *Plasmodium relictum* [18], as did Clade B, which matched the Hawaiian *P. relictum* strain GRW4 [33]. An additional *P. relictum* matched samples falling in Clade E [34]. Clade I matched a lineage described as *P. elongatum* [18].

*Haemoproteus* clades displayed greater host discrepancy and lineages branched in an apparent pattern of separation according to host family infected (Fig. 4b). Clades J and K are closely related to lineages described as *Haemoproteus sylvae* [35], whilst Clade M contained a lineage described as *H. lanii* [36]. Clades Q and R were the most common *Haemoproteus* lineages, with both lineages infecting birds almost exclusively from the Ploceidae. Clades P and Q matched lineages in the MalAvi database described as VIMWE1 [19] and REB2 [37] respectively. Clades R and S were closely related to lineages described as RBQ01 [38].

### Host Specificity

**Figure 5:**
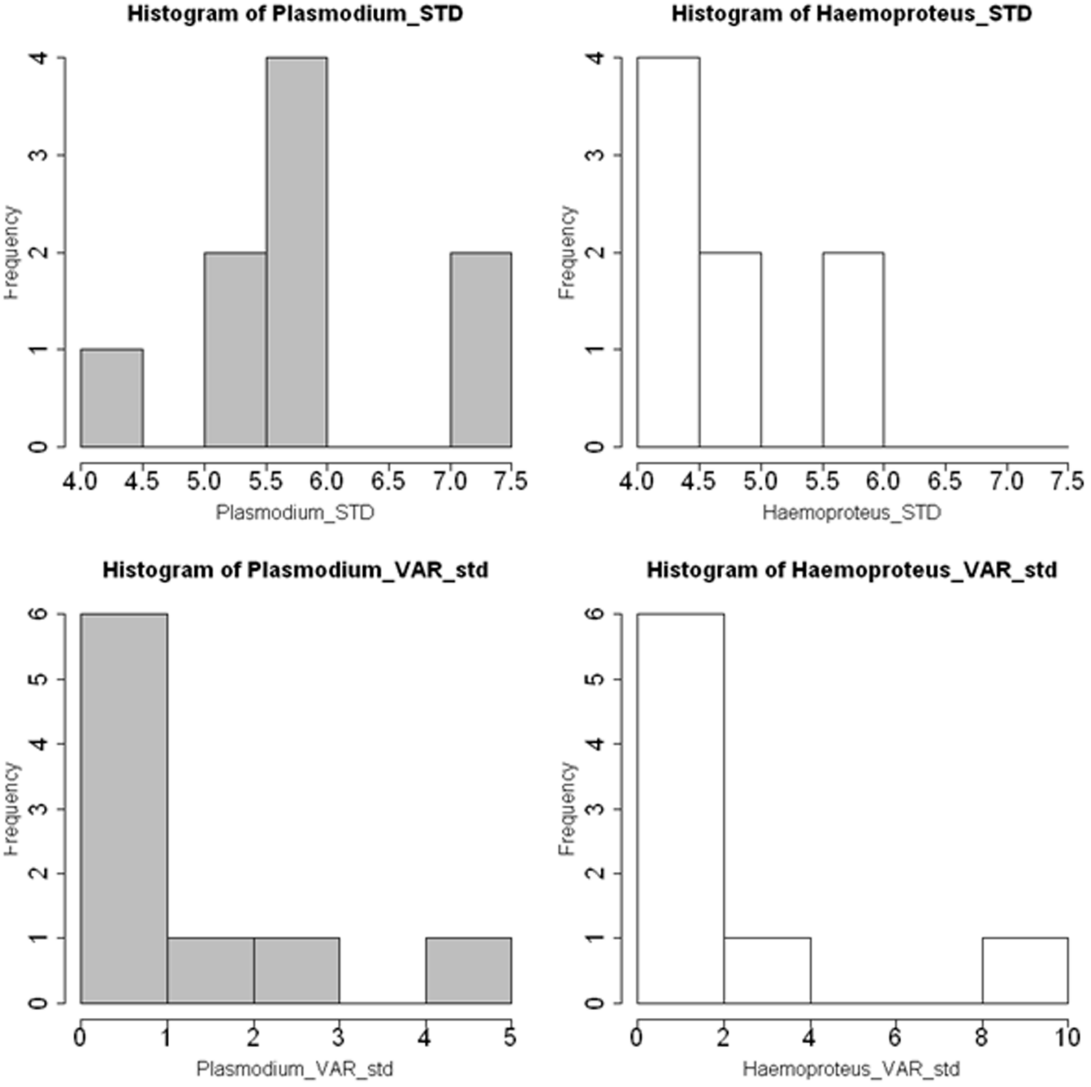
Phylogenetic distance and diversity between host species infected by *Plasmodium* and *Haemoproteus* spp.

*Plasmodium* lineages infected a greater diversity of host species, displaying higher overall S_TD_ values (mean = 5.78; s.d. = 6.99) than *Haemoproteus* lineages (mean = 4.68; s.d. = 6.76). S_TD_ values for the majority of *Plasmodium* lineages scored between 5.5 and 6.0, compared to 4.0–4.5 for *Haemoproteus* lineages (Fig. 5). Overall the taxonomic diversity scores (VAR_STD_) for both *Plasmodium* and *Haemoproteus* lineages were similar, with most lineages ranging between 0 and 1. However, a notable exception occurred amongst *Haemoproteus* lineages, where one lineage (Clade N) had the highest VAR_STD_ value of 8.44 (Fig. 5).

## Discussion

### Overall Infection Prevalence

*Plasmodium* spp accounted for the greatest prevalence of infections among wetland passerines, with *Plasmodium* infections being generally widespread and the sole cause of infection in five avian families (Fig. 2). The overall *Plasmodium* infection prevalence (17%) and prevalence range (3–25%) were lower than that found in West African birds (45%; [39]), which also had a much wider prevalence range (1–69%). However, *Plasmodium* prevalence was higher than infection rates in North American passerines (3–7%; [40],[41]). Otherwise, prevalence rates were consistent with those reported in other studies: viz 1–24% *Plasmodium* infection rates in European passerines [42],[43] and 3–35% infection rates in Asian passerines [44]. The ploceid family had heavier infections than would be found by chance (Table 3); Southern Red Bishops and Cape Weavers were prominent hosts of both *Plasmodium* and *Haemoproteus*, and were significantly more heavily infected than other avian families. The prevalence in ploceids (27%) was higher than the 21% reported from birds sampled in the neighbouring Eastern Cape [45]. *Plasmodium* infection was also notable in Motacillidae (wagtails) and Laniidae (shrikes), which have previously featured as prominently infected hosts in avian malaria studies conducted in East and West Africa [39],[46].

*Haemoproteus* prevalence [4%] was significantly lower than that of *Plasmodium*, with *Haemoproteus* strains also infecting a narrower range of host families (Fig. 3). Similarly, *Haemoproteus* prevalence was lower than rates of 13–22% reported elsewhere in sub-Saharan Africa [39],[47], and rates of 6–14% in Asian and South American passerines [48],[49]. European passerines were the only population exhibiting comparable *Haemoproteus* prevalences of 4–11% [50],[51].

### Lineage Infection Patterns

Lineages in *Plasmodium* clades A and C were the most common infections observed: they occurred predominantly in weavers but also in a wide range of other host families. Both clades seem to be endemic to southern Africa, with matching GenBank lineages isolated previously from gannets, weavers and canaries in the region [18]. Branching relationships in the *Plasmodium* tree (Fig. 4a) and matching lineages identify both clades as varying strains of *P. relictum* [18],[33]. Although Clade A infected mostly weavers, indications are that both Clades A and C are generalist in nature, with a wide diversity of host species infected by both. Additionally, *P. relictum* is known as a generalist species, having been isolated from more than 200 different bird species worldwide [21]. Clades A and C may represent some of the more established lineages in this region, and their predominance could be an indicator of their adaptation to resident birds, vectors, or environments [52],[53]. Previous molecular studies on *Plasmodium* spp. in the region [47], have also reported birds infected with the same *Plasmodium* strain as that occurring in Clade H (RFF1).

Matching *Plasmodium* lineages (P18 and KU81) isolated from non-passerine hosts further highlight the diversity in host choice exhibited by *Plasmodium* lineages generally and may explain why it thrives in an environment rich in biodiversity [6]. Exceptions to the generalist trend occurred mainly in lineages that were not originally isolated from Africa, such as Clades B (GRW4), E (SGS1), F (ADP-2011) and I (P52). Matching GenBank strains in these clades also originated from different host species to those sampled in this study [33],[54],[60], suggesting that these clades may also be generalists, but with a restricted prevalence in the region. Restrictions may be due to recent regional establishment or isolated occurrences via a migratory host. For example, restricted prevalence has occurred in passerines introduced from Europe, which were infected with different *Plasmodium* species than those found locally [55]. Alternatively, factors such as competition from endemic lineages infecting the same host species, or host-related factors such as age and territory size, may potentially influence the amplification of one parasite lineage over another [56].

*Haemoproteus* spp. showed more fidelity with respect to host family groups and were the predominant cause of infection in sparrows, shrikes and white-eyes—mono-specific infections occurred in the latter two families. *Haemoproteus lanii* [36] in Clade M was the principal infection in shrikes, which is consistent with shrikes being the principal host family for this parasite species [21]. Other *Haemoproteus* spp. have also been reported from shrikes regionally [22],[57], suggesting that shrikes may be host to additional specialists. Host specificity in *Haemoproteus* spp. was also apparent in the phylogenetic relationships of various *Haemoproteus* strains: for example, Clades P, Q and S infected weavers almost exclusively (Fig. 5b). Clade S was previously isolated from red-billed queleas (*Quelea quelea*) in sub-saharan Africa [38]; queleas are also members of the weaver family [31]. Clades P and Q have been previously isolated from passerines in sub-saharan Africa only [19],[37]. The host species fidelity displayed by lineages P to S suggests that there may have been geographical restrictions for these lineages; possibly stemming from host availability and co-evolutionary restraints [58]. The majority of matching *Haemoproteus* lineages also occurred in endemic host species [9],[60], which was in contrast to *Plasmodium* strains. However this was not the case for all *Haemoproteus* lineages; several were isolated from non-African hosts with host geographic distribution appearing not to restrict host fidelity tendencies – although these hosts invariably came from the same host family as their counterparts in the region. For example, four *Haemoproteus* lineages (SYAT7; SYAT26; GRW1; 745) closely related to Clades J and K, were repeatedly isolated from Eurasian Blackcaps (*Sylvia atricapilla*) and Great Reed Warblers (*Acrocephalus arundinaceus*), both of which are Palearctic-breeding warbler species [35],[59]. Similarly, Clade L (LIN18) was isolated predominantly from white-eyes and also occurred in white-eyes from the Comoros and Madagascar [19]. Therefore results here indicate that *Haemoproteus* lineages may have developed host specificity tendencies potentially as a result of both cospeciation with specific host species, as well as with geographic factors.

### Host Specificity Index

S_TD_ scores in this study appeared to be the more definitive indicator of host specificity between *Plasmodium* and *Haemoproteus* lineages. The higher overall S_TD_ scores for *Plasmodium* spp. was in concurrence with the larger number of host species in the study infected with *Plasmodium* lineages (12 avian families represented); compared with *Haemoproteus* (8 families). We also found that *Plasmodium* clades with the highest prevalence (Clades A and C) displayed a high prevalence of mono-specific infections (Fig. 4a), which concurred with host infection patterns seen in similar avian haemosporidian studies [15]. Higher S_TD_ scores generally indicated greater phylogenetic distances between host species infected by *Plasmodium* lineages. *Plasmodium* spp. also registered low VAR_STD_ values, further indicating high taxonomic variety between hosts [8]. Although the majority of *Haemoproteus* lineages displayed similarly low VAR_STD_ values, the range in values was greater than for *Plasmodium* spp (Fig. 5). As described by Poulin and Mouillot [8], a high VAR_STD_ value can arise when several infected host species are congeners, but one or two remain taxonomically distinct. This was the case for *Haemoproteus* Clades L and N (Fig. 4b), which infected several closely related host species as well as one or two taxonomically distinct species. Also, the lower S_TD_ scores for *Haemoproteus* spp. suggested that infected host species were more closely related to each other

Avian malaria parasites have a demonstrated ability to ‘host-switch’, and/or to diverge and form sub-species within a single host species, regardless of geographic restrictions or range expansion to novel hosts [56],[60]. These events may explain the closely related derivatives of *P. relictum* seen in clades A to C, in *Haemoproteus* clades J and K, and in other closely related lineages which to date, have only been isolated from warblers [61]. Parasites infected a wide range of hosts although the prevalence of non-endemic *Plasmodium* strains appeared restricted. By contrast, prevalence of *Haemoproteus* lineages seemed very much host-driven, and comparable work on *Haemoproteus* spp. elsewhere [4],[19] supports the likelihood that host specialization is the ‘*modus operandi*’ for this genus. *Haemoproteus* infection patterns also adhered to trends of host specificity as outlined by Poulin and Mouillot [8], particularly to the genus level (Fig. 4b). *Plasmodium* spp. by contrast, exhibited more plasticity between specialist and generalist tendencies, and also exhibited a high prevalence in mono-specific hosts, a trend also in keeping with previous findings [13],[15].

Several outcomes presented here strongly support previous works demonstrating the elastic host ranges of avian haemosporidia. Although this study was conducted in a comparatively small region of sub-saharan Africa, it is remarkable that the general patterns of host-parasite interaction in *Plasmodium* and *Haemoproteus* genera remain prominent, regardless of geographic locality or host species endemicity. Although many lineages of avian haemosporidia have a wide regional and inter-continental distribution, the number apparently occurring in the Western Cape alone, together with additional novel strains, hints at a strikingly high level of lineage diversity within and between avian haemosporidian species in general. It also further attests to the efficacy of birds as dispersers of disease, as well as ideal hosts for novel parasites [62]. Molecular technology has substantially expanded our capacity to detect avian haemosporidia to species and sub-species level. It is increasingly becoming apparent that a species description can apply to several lineages, rendering traditional species names as more of a guideline than an empirical measure of haemosporidian parasite diversity. This can often lead to some confusion and ambiguity, and has prompted discussion as to how to rectify the situation [63].

Most of the birds that were infected with *Plasmodium* came from just five family groups, suggesting that *Plasmodium* prevalence patterns may correlate with the abundance of these hosts. These five passerine families also appear to be promising candidates for further investigation of host-parasite cospeciation patterns in avian malaria ecology.
