# Correction: Host Specificity And Co-Speciation In Avian Haemosporidia In The Western Cape, South Africa

**DOI:** 10.1371/journal.pone.0129188

**Published:** 2015-05-20

**Authors:** Sharon Okanga, Graeme S. Cumming, Philip A. R. Hockey, Lisa Nupen, Jeffrey L. Peters

An affiliation is missing for the fourth author. Lisa Nupen is affiliated with #1 and with #3 National Zoological Gardens of South Africa, Pretoria, Gauteng, South Africa.
